# Development of neonatal brain functional centrality and alterations associated with preterm birth

**DOI:** 10.1093/cercor/bhac444

**Published:** 2022-11-20

**Authors:** Sunniva Fenn-Moltu, Sean P Fitzgibbon, Judit Ciarrusta, Michael Eyre, Lucilio Cordero-Grande, Andrew Chew, Shona Falconer, Oliver Gale-Grant, Nicholas Harper, Ralica Dimitrova, Katy Vecchiato, Daphna Fenchel, Ayesha Javed, Megan Earl, Anthony N Price, Emer Hughes, Eugene P Duff, Jonathan O’Muircheartaigh, Chiara Nosarti, Tomoki Arichi, Daniel Rueckert, Serena Counsell, Joseph V Hajnal, A David Edwards, Grainne McAlonan, Dafnis Batalle

**Affiliations:** Department of Forensic and Neurodevelopmental Sciences, Institute of Psychiatry, Psychology & Neuroscience, King’s College London, London, SE5 8AF, United Kingdom; Centre for the Developing Brain, School of Biomedical Engineering & Imaging Sciences, King's College London, London, SE1 7EH, United Kingdom; Wellcome Centre for Integrative Neuroimaging (WIN FMRIB), University of Oxford, Oxford, OX3 9DU, United Kingdom; Department of Forensic and Neurodevelopmental Sciences, Institute of Psychiatry, Psychology & Neuroscience, King’s College London, London, SE5 8AF, United Kingdom; Centre for the Developing Brain, School of Biomedical Engineering & Imaging Sciences, King's College London, London, SE1 7EH, United Kingdom; Centre for the Developing Brain, School of Biomedical Engineering & Imaging Sciences, King's College London, London, SE1 7EH, United Kingdom; Centre for the Developing Brain, School of Biomedical Engineering & Imaging Sciences, King's College London, London, SE1 7EH, United Kingdom; Biomedical Image Technologies, ETSI Telecomunicación, Universidad Politécnica de Madrid & CIBER-BBN, Madrid, 28040, Spain; Centre for the Developing Brain, School of Biomedical Engineering & Imaging Sciences, King's College London, London, SE1 7EH, United Kingdom; Centre for the Developing Brain, School of Biomedical Engineering & Imaging Sciences, King's College London, London, SE1 7EH, United Kingdom; Department of Forensic and Neurodevelopmental Sciences, Institute of Psychiatry, Psychology & Neuroscience, King’s College London, London, SE5 8AF, United Kingdom; Centre for the Developing Brain, School of Biomedical Engineering & Imaging Sciences, King's College London, London, SE1 7EH, United Kingdom; MRC Centre for Neurodevelopmental Disorders, King’s College London, London, SE1 1UL, United Kingdom; Centre for the Developing Brain, School of Biomedical Engineering & Imaging Sciences, King's College London, London, SE1 7EH, United Kingdom; Department of Forensic and Neurodevelopmental Sciences, Institute of Psychiatry, Psychology & Neuroscience, King’s College London, London, SE5 8AF, United Kingdom; Centre for the Developing Brain, School of Biomedical Engineering & Imaging Sciences, King's College London, London, SE1 7EH, United Kingdom; Department of Forensic and Neurodevelopmental Sciences, Institute of Psychiatry, Psychology & Neuroscience, King’s College London, London, SE5 8AF, United Kingdom; Centre for the Developing Brain, School of Biomedical Engineering & Imaging Sciences, King's College London, London, SE1 7EH, United Kingdom; Department of Forensic and Neurodevelopmental Sciences, Institute of Psychiatry, Psychology & Neuroscience, King’s College London, London, SE5 8AF, United Kingdom; Centre for the Developing Brain, School of Biomedical Engineering & Imaging Sciences, King's College London, London, SE1 7EH, United Kingdom; MRC Centre for Neurodevelopmental Disorders, King’s College London, London, SE1 1UL, United Kingdom; Department of Forensic and Neurodevelopmental Sciences, Institute of Psychiatry, Psychology & Neuroscience, King’s College London, London, SE5 8AF, United Kingdom; Centre for the Developing Brain, School of Biomedical Engineering & Imaging Sciences, King's College London, London, SE1 7EH, United Kingdom; Department of Forensic and Neurodevelopmental Sciences, Institute of Psychiatry, Psychology & Neuroscience, King’s College London, London, SE5 8AF, United Kingdom; Centre for the Developing Brain, School of Biomedical Engineering & Imaging Sciences, King's College London, London, SE1 7EH, United Kingdom; Paediatric Liver, GI and Nutrition Centre and MowatLabs, King’s College London, London, SE5 9RS, United Kingdom; Centre for the Developing Brain, School of Biomedical Engineering & Imaging Sciences, King's College London, London, SE1 7EH, United Kingdom; Centre for the Developing Brain, School of Biomedical Engineering & Imaging Sciences, King's College London, London, SE1 7EH, United Kingdom; Wellcome Centre for Integrative Neuroimaging (WIN FMRIB), University of Oxford, Oxford, OX3 9DU, United Kingdom; Department of Paediatrics, University of Oxford, Oxford, OX3 9DU, United Kingdom; Department of Forensic and Neurodevelopmental Sciences, Institute of Psychiatry, Psychology & Neuroscience, King’s College London, London, SE5 8AF, United Kingdom; Centre for the Developing Brain, School of Biomedical Engineering & Imaging Sciences, King's College London, London, SE1 7EH, United Kingdom; MRC Centre for Neurodevelopmental Disorders, King’s College London, London, SE1 1UL, United Kingdom; Centre for the Developing Brain, School of Biomedical Engineering & Imaging Sciences, King's College London, London, SE1 7EH, United Kingdom; Department of Child and Adolescent Psychiatry, Institute of Psychiatry Psychology and Neuroscience, King’s College London, London, SE5 8AF, United Kingdom; Centre for the Developing Brain, School of Biomedical Engineering & Imaging Sciences, King's College London, London, SE1 7EH, United Kingdom; MRC Centre for Neurodevelopmental Disorders, King’s College London, London, SE1 1UL, United Kingdom; Paediatric Neurosciences, Evelina London Children’s Hospital, Guy’s and St Thomas’ NHS Foundation Trust, London, SE1 7EH, United Kingdom; Department of Bioengineering, Imperial College London, London, SW7 2AZ, United Kingdom; Biomedical Image Analysis Group, Imperial College London, London, SW7 2AZ, United Kingdom; Institute for AI and Informatics in Medicine, Klinikum rechts der Isar, Technical University of Munich, Munich, 81675, Germany; Centre for the Developing Brain, School of Biomedical Engineering & Imaging Sciences, King's College London, London, SE1 7EH, United Kingdom; Centre for the Developing Brain, School of Biomedical Engineering & Imaging Sciences, King's College London, London, SE1 7EH, United Kingdom; Centre for the Developing Brain, School of Biomedical Engineering & Imaging Sciences, King's College London, London, SE1 7EH, United Kingdom; MRC Centre for Neurodevelopmental Disorders, King’s College London, London, SE1 1UL, United Kingdom; Department of Forensic and Neurodevelopmental Sciences, Institute of Psychiatry, Psychology & Neuroscience, King’s College London, London, SE5 8AF, United Kingdom; MRC Centre for Neurodevelopmental Disorders, King’s College London, London, SE1 1UL, United Kingdom; Department of Forensic and Neurodevelopmental Sciences, Institute of Psychiatry, Psychology & Neuroscience, King’s College London, London, SE5 8AF, United Kingdom; Centre for the Developing Brain, School of Biomedical Engineering & Imaging Sciences, King's College London, London, SE1 7EH, United Kingdom

**Keywords:** brain development, functional centrality, resting-state connectivity, preterm birth, neonatal

## Abstract

Formation of the functional connectome in early life underpins future learning and behavior. However, our understanding of how the functional organization of brain regions into interconnected hubs (centrality) matures in the early postnatal period is limited, especially in response to factors associated with adverse neurodevelopmental outcomes such as preterm birth. We characterized voxel-wise functional centrality (weighted degree) in 366 neonates from the Developing Human Connectome Project. We tested the hypothesis that functional centrality matures with age at scan in term-born babies and is disrupted by preterm birth. Finally, we asked whether neonatal functional centrality predicts general neurodevelopmental outcomes at 18 months. We report an age-related increase in functional centrality predominantly within visual regions and a decrease within the motor and auditory regions in term-born infants. Preterm-born infants scanned at term equivalent age had higher functional centrality predominantly within visual regions and lower measures in motor regions. Functional centrality was not related to outcome at 18 months old. Thus, preterm birth appears to affect functional centrality in regions undergoing substantial development during the perinatal period. Our work raises the question of whether these alterations are adaptive or disruptive and whether they predict neurodevelopmental characteristics that are more subtle or emerge later in life.

## Introduction

The brain is continuously changing across the lifespan but is most plastic and modifiable during early life (De Asis-Cruz et al. 2015). Following the establishment of its general architecture during the first 2 trimesters of gestation, the third trimester sees vast axonal growth and rapid expansion of the cortex and the start of myelination, synaptogenesis, and dendritic formation, which continues into the postnatal period (see review by Keunen et al. 2017). Alongside the establishment of the brain’s structural architecture, the formation and differentiation of functional circuits are also taking place (Cao et al. 2017). With such complex processes occurring during late gestation, the brain may be particularly vulnerable to factors disrupting typical maturational trajectories in this timeframe, such as preterm birth.

Preterm birth (<37 weeks’ gestation) most commonly happens during the third trimester and is estimated to affect around 11% of all live births around the world (Chawanpaiboon et al. 2019). Although improvements in perinatal care have increased survival rates, preterm infants still show higher incidence of neurodevelopmental conditions such as autism and attention deficit hyperactivity disorder (ADHD; Wang et al. 2017; Bröring et al. 2018) and related neurodevelopmental complications in later childhood such as language delay and altered socio-emotional and behavioral functioning (Boardman and Counsell 2020). Such difficulties further translate into a significant impact on the quality of life indicators such as academic achievement and peer relationships (Rogers et al. 2018). Therefore, studying both typical and atypical functional connectivity during the perinatal period may help us identify potential early biological underpinnings of developmental and behavioral phenotypes observed in preterm-born children as they grow.

The graph-theoretical modeling framework has provided an important insight into the topological architecture of the neonatal brain. Both the structural and functional brain networks show highly efficient connectivity patterns, with structural network development preceding the development of functional network architecture. The networks develop in a primary-to-higher-order ontogenetic sequence and establish a pattern of functional connectivity characterized by both segregation and integration (Cao et al. 2017). For a comprehensive review of graph theoretical modeling of the neonatal connectome, see Zhao et al. (2019). Here, we employ the graph-theory-based network measure voxel-wise degree centrality (DC) to resting-state functional magnetic imaging (rs-fMRI) data. DC maps the functional connectome architecture at the voxel level by measuring the number of direct connections between a given voxel and the rest of the brain (De Asis-Cruz et al. 2015; Gozdas et al. 2018). This provides an insight into the patterns of functional connectivity within the whole brain network (Zuo et al. 2012). This approach does not require a priori definition of regions of interest (ROIs), and does not require a parcellation step, which has proven challenging in neonatal imaging (Oishi et al. 2019). As the DC of a voxel reflects the relative importance of that voxel within the network, it allows us to identify regions which are “central” (“functional hubs”) and thus of likely functional importance. DC has been shown to have relatively high test–retest reliability in adults (Zuo and Xing 2014) and has offered important insight into the complexity of the adult functional connectome (Zuo et al. 2012). While other network measures are available, such as participation coefficient and within module-z (Power et al. 2013), we chose weighted degree as this measure has been employed both in the study of typical function (van den Heuvel and Sporns 2013) and has recently been shown to be a reproducible metric to detect atypical patterns of centrality associated with relevant neurodevelopmental disorders such as autism (Holiga et al. 2019) for which preterm birth is a known risk factor (Crump et al. 2021). However, studies of already diagnosed individuals, even in later childhood, cannot untangle the effect of early exposure and the secondary and/or compensatory effects of living with neurodevelopmental difficulties on the functional connectome. Studies in the earlier postnatal period are needed to understand how exposures in this period shape the developing brain.

Initial work in infants has shown that the neonatal functional connectome shows a heavy-tailed degree distribution (De Asis-Cruz et al. 2015; Oldham and Fornito 2019), with the most highly connected, and thus putative “hub” regions, located within motor, sensory, auditory, and the visual cortices (Fransson et al. 2011). Efforts have also been made to understand how functional DC changes throughout the perinatal period and how it is impacted by preterm birth. For example, neonates scanned shortly after birth between 31.3 and 41.7 weeks of postmenstrual age (PMA) (Cao et al. 2017) showed age-related increases in DC predominantly within the precentral and postcentral gyri and supplementary motor area and decreases within the posterior cingulate and precuneus cortex. However, preterm-born infants were not compared to term-born infants scanned at an equivalent age, therefore the results capture the effect of gestational age (GA) rather than the impact of preterm birth. Only one small study has examined preterm (≤31 weeks’ gestation) and term neonates with both groups scanned at term-equivalent age. The authors reported that, compared to full-term-born infants, very preterm infants had higher DC in the auditory and language networks compared to term infants, whereas term infants had higher centrality in frontal networks compared to very preterm infants (Gozdas et al. 2018). Moreover, the functional connectome was not examined at voxel-level resolution, which may be more sensitive to the subtle alterations caused by preterm birth. Finally, no study has investigated whether the functional centrality in the perinatal period is altered by preterm birth and whether this has developmental consequences later in infancy.

The Developing Human Connectome Project (dHCP) represents the largest open-source sample of rs-fMRI neonatal data with high spatial and temporal resolution (Edwards et al. 2022), thus providing a unique opportunity to characterize the functional centrality during this early phase of life. In this study, we leverage dHCP data to characterize the functional centrality in the neonatal brain at the voxel level in term-born and preterm-born neonates. We subsequently explore the relationship between neonatal functional centrality and behavioral outcome measures at 18 months of age. By understanding how functional connectivity patterns typically develop with age (PMA at scan) and how typical development is affected by the degree of preterm birth (GA at birth), we can both establish a typical maturation profile and investigate the consequence of preterm birth. Finally, we assessed whether the functional centrality in early life is predictive of general neurodevelopmental outcomes in toddlerhood in term and preterm-born children.

## Methods

### Participants

Research participants were recruited as part of the dHCP (http://www.developingconnectome.org/). Ethical approval was given by the UK National Research Ethics Authority (14/LO/1169), and written consent was obtained from all participating families prior to data collection. For the purpose of this study, we included subjects without visible abnormality on magnetic resonance imaging of possible/likely clinical significance following reporting by an experienced neonatal neuroradiologist. Findings leading to exclusion were major lesions within white matter, cortex, cerebellum and/or basal ganglia, small head/brain <first centile, and isolated nonbrain anomalies with possible clinical significance. Incidental findings with unlikely significance for clinical outcome were not excluded, such as subdural hemorrhages, isolated subependymal cysts, and punctate white matter lesions. In the case of a multiple pregnancy, only 1 sibling was included in the subsequent analysis. Subjects were also excluded due to excessive in-scanner motion (>10% framewise displacement [FD] motion outliers [FD > 1.5IQR + 75th centile]). The resulting population was 366 neonates. Of these, 300 were born at term and 66 were born preterm, i.e. before 37 weeks’ gestation. Very preterm-born (<32 weeks GA at birth) and extremely preterm-born (<28 weeks GA at birth) neonates represented 17/66 (26%) and 8/66 (12%) of the total preterm population. Scans were acquired at term or term-equivalent age, respectively. See the demographic and clinical details in Table 1. For analyses assessing the effect of PMA at scan on DC, only term-born infants were included (*n* = 300). The effect of preterm birth was assessed by treating GA at birth as a binary variable, with comparisons carried out between the neonates born <37 weeks (preterm, *n* = 66) and those born at >37 weeks (term, *n* = 300). As the <37 week cut-off for preterm-birth is somewhat arbitrary (Kramer et al. 2012; Vogel et al. 2018), in addition to the dichotomous analysis, term and preterm groups were also combined (*n* = 366), and GA at birth was treated as a continuous variable.

**Table 1:** Subject demographics, clinical details and follow up. Continuous variables expressed as mean and SD. Categorical variables expressed as number and percentage.

Group	Term (i)	Preterm (ii)	Test statistic	*P*
	(*n* = 300)	(*n* = 66)		
Demographics & clinical details				
GA at birth (weeks)	40.06 (1.28)	32.70 (3.36)	12.73*^a^*	*P* < 0.001
PMA at scan (weeks)	41.54 (1.67)	40.50 (2.16)	3.67*^a^*	*P* < 0.001
Sex (number of males)	158 (52.67%)	39 (59.1%)	0.90*^b^*	*P* = 0.343
% FD outliers	5.46 (2.60)	4.48 (2.54)	2.81*^a^*	*P* = 0.005
No incidental findings	179 (59.7%)	18 (27.3%)	22.84*^b^*	*P* < 0.001
Follow up				
Corrected age at follow-up (months)^c^	18.92 (1.78)	19.37 (3.26)	−0.17^a^	*P* = 0.870
Uncorrected age at follow-up (months)^c^	18.90 (1.78)	21.04 (3.38)	−7.81^a^	*P* < 0.001
Bayley cognition (composite score)^c^	102.03 (10.52)	99.50 (13.02)	1.18^a^	*P* = 0.238
Bayley language (composite score)^c^	99.63 (15.46)	95.46 (15.57)	1.43^a^	*P* = 0.154
Bayley motor (composite score)^c^	102.55 (9.83)	99.60 (10.05)	2.09^a^	*P* = 0.036
Q-CHAT (total score)^d^	29.70 (8.28)	31.40 (11.63)	−0.62^a^	*P* = 0.539
CBCL Externalizing score^e^	11.43 (6.82)	9.92 (7.30)	1.57^a^	*P* = 0.116
CBCL Internalizing score^e^	5.51 (4.88)	5.33 (5.09)	0.47^a^	*P* = 0.637
CBCL Other Behavioral Problems score^e^	7.80 (4.72)	7.75 (5.02)	0.10^a^	*P* = 0.921

^a^ *Z* (Mann Whitney U-test).

^*^b^*^ *X^2^* test.

^*^c^*^Bayley (Bayley Scales of Infant Development: Third Edition) number of completed assessments: 241 term, 50 preterm.

^*^d^*^Q-CHAT number of completed assessments: 239 term, 48 preterm.

^*^e^*^CBCL number of completed assessments: 238 term, 48 preterm.

### Image acquisition

Infants were scanned at the Evelina Newborn Imaging Centre, Evelina London Children’s Hospital using a 3 Tesla Philips Achieva system (Best, NL) and a neonatal 32 channel receive head coil and imaging system (Rapid Biomedical GmBH, DE) (Hughes et al. 2017). Images were acquired during natural sleep following feeding, swaddling, and immobilization in a vacuum-evacuated mattress (Med-Vac, CFI Medical Solutions, Fenton, MI, United States). Hearing protection (molded dental putty [President Putty, Coltene Whaledent, Mahwah, NJ, United States]) in addition to adhesive earmuffs (MiniMuffs, Natus Medical, Inc., San Carlos, CA, United States) and physiological monitoring devices (oxygen saturations, heart rate, and axillary temperature) were applied before image acquisition, and all scans were attended by a neonatal nurse and/or pediatrician. Blood oxygen level–dependent (BOLD) fMRI data were acquired using a multislice gradient-echo echo planar imaging sequence with multiband excitation (multiband factor 9) with the following parameters: repetition time, 392 ms; echo time, 38 ms; spatial resolution, 2.15 mm isotropic; flip angle, 34°; 45 slices; total time, 15 min 3 s (2,300 volumes). High-resolution anatomical T1-weighted images (reconstructed spatial resolution, 0.8 mm isotropic; field of view, 145 × 122 × 100 mm^3^; and repetition time, 4,795 ms) and T2-weighted images (reconstructed spatial resolution, 0.8 mm isotropic; field of view, 145 × 145 × 108 mm; repetition time, 12 s; and echo time, 156 ms) were acquired for registration and clinical reporting (Edwards et al. 2022).

### Functional data preprocessing

The data were preprocessed using a pipeline optimized for neonatal imaging as part of the dHCP (Fitzgibbon et al. 2020). In brief, this included dynamic distortion and intra- and intervolume motion correction using a pipeline including slice-to-volume and rigid-body registration. Motion, cardiorespiratory, and multiband acquisition artifacts were regressed out together with single-subject ICA noise components identified using FSL FIX (Griffanti et al. 2014; Salimi-Khorshidi et al. 2014). Data were subsequently registered into T2w native space using boundary-based registration and were nonlinearly transformed to a 40-week neonatal template from the extended dHCP volumetric atlas (Schuh et al. 2018). Participants with high FD (FD > 1.5IQR + 75th centile) in >230 (10%) of the 2,300 volumes were excluded from analysis. The data were subsequently smoothed with a 3-mm Gaussian smoothing kernel, band-pass-filtered at 0.01–0.1 Hz and were masked with a gray matter mask defined in template space, consisting of 45,110 voxels.

### Functional data analysis

Graphs were constructed from the data at a voxel level. The BOLD time series for each voxel within the predefined gray matter mask was extracted, and a correlation matrix was made using the temporal Pearson’s correlation of time series between each pair of voxels. A threshold of 0.20 was used to avoid including spurious connections in the correlation matrix (Buckner et al. 2009; Holiga et al. 2019) and only positive connections were assessed (Murphy et al. 2009). To quantify the nodal centrality for the graphs constructed from the rs-fMRI data, we used weighted DC (nodal strength). For a weighted graph, weighted DC is defined as the sum of the weights from edges connecting to a node. Thus, a node has a high degree if it is strongly connected to many other nodes.

The network analysis was repeated using no threshold for positive connections (i.e. >0.0) and a proportional threshold of 10% to determine whether the results were affected by threshold choice.

Weighted DC was calculated at the individual level, and the voxel-wise maps were standardized to *z*-scores to allow comparisons across subjects and were used as the inputs of the subsequent group-level analysis (hereby referred to as DC, if not otherwise indicated). Hence, these DC values are relative and reflect centrality relative to the rest of the brain rather than absolute centrality. In order to account for the differences in network cost influencing network topology (Achard and Bullmore 2007), network density (i.e. the ratio between edges present in the network graph and the maximum number of edges that the graph can contain) was calculated for each subject to be included as a covariate in analyses. An average functional brain network was also constructed by converting each subject’s thresholded connectivity matrix into a Fisher Z matrix and averaging across participants. The averaged Fisher Z matrices were then backtransformed by using the inverse Fisher Z-transformation. DC was subsequently calculated on the average matrix. Using the same approach, weekly average matrices were also constructed from 38 to 41 weeks of PMA. To ensure consistency across weeks, the same number of subjects were selected for each week (15 infants), with the lowest number of postnatal days (PND) as the selection criteria.

### Follow-up outcome data

Neurodevelopmental follow-up assessments were offered to all participants at around 18 months corrected age as part of the dHCP. Neurodevelopment was assessed with a number of behavioral and observational assessments, including the Bayley Scale of Infant and Toddler Development, Third Edition (Bayley 2006), the Child Behavior Checklist 1^1/2^-5 (CBCL; Achenbach and Rescorla 2000), and the Quantitative Checklist for Autism in Toddlers (Q-CHAT; (Allison et al. 2008). The cognitive, motor, and language composite scores of the Bayley Scale; the Externalizing, Internalizing, and Other Behavioral Problems raw scores of the CBCL, and the total Q-CHAT score were included as the outcome measures in this study. Index of multiple deprivation (IMD) was included as a covariate in statistical analyses. The IMD score is a composite score of social risk calculated based on the mother’s address at the time of birth (Abel et al. 2016). As not all infants completed all follow-up assessments, the number of infants with data available for each measure is indicated in Table 1. Assessments were performed at St. Thomas’ Hospital, London, by 2 experienced assessors (authors AC, pediatrician, and SF, chartered psychologist).

### Statistical analyses

In the term infant group, the effect of PMA on DC was explored in a general linear model (GLM), including PND, sex, network density, total number of FD outliers, and voxel-wise temporal signal-to-noise ratio (tSNR) as covariates (*y* ~ β_0_ + β_1_PMA + β_2_PND + β_3_ Sex + β_4_Density + β_5_FD + β_6_tSNR). A groupwise comparison was conducted between preterm and term infants in a GLM, including PMA at scan, sex, network density, total number of FD outliers, and tSNR as covariates (*y* ~ β_0_ + β_1_Birth Status + β_2_PMA + β_3_Sex + β_4_Density + β_5_FD + β_6_tSNR). The effect of GA at birth as a continuous variable was also explored in a GLM with the same covariates as in the groupwise analysis (*y* ~ β_0_ + β_1_GA + β_2_PMA + β_3_Sex + β_4_Density + β_5_FD + β_6_tSNR). A GLM assessing the interaction between group (term vs. preterm) and PMA was also run to assess whether the effect of PMA at scan on DC varies as a function of preterm birth (*y* ~ β_0_ + β_1_Birth Status + β_2_PMA + β_1_Birth Status* β_2_PMA + β_3_Sex + β_4_Density + β_5_FD β_6_tSNR). Network density and tSNR were included as covariates because subjects have varying network density (0.58 ± 0.15 [mean ± standard deviation {SD}]), and the within-subject distribution of tSNR in the neonatal fMRI scans is not uniformly distributed. FD outliers were included as a covariate as head motion during fMRI data acquisition is known to cause signal artifact and affect the estimates of functional connectivity (Satterthwaite et al. 2013). A version of the standard anatomical automatic labeling atlas (Tzourio-Mazoyer et al. 2002) adapted to the neonatal brain (Shi et al. 2011) was used for the localization of results. Statistical analyses were carried out using nonparametric permutation inference as implemented in the FSL randomise tool, with 10,000 permutations per test (Winkler et al. 2014). Threshold-free cluster enhancement and family-wise error rate were applied to correct for voxel-wise multiple comparisons (Smith and Nichols 2009).

The effects of PMA at scan and GA at birth were also explored within functional resting-state networks (RSNs) previously identified in the neonatal brain (Eyre et al. 2021). The RSNs were nonlinearly registered using ANTs SyN (Avants et al. 2008) from the release 2 dHCP atlas week-40 volumetric template space to the dHCP extended atlas week-40 volumetric template space (Schuh et al. 2018). Within the RSNs, median DC (*z*-score) was extracted, and partial correlations were conducted between median DC and PMA at scan (term-born neonates, controlling for GA at birth, sex, total number of FD outliers, and network density). In a multivariate model, the relationship between birth status (preterm vs. term-born neonates) and median DC in the RSNs was assessed with PMA at scan, sex, FD outliers, and network density as covariates (*y* ~ β_0_ + β_1_Birth Status + β_2_PMA + β_3_Sex + β_4_Density + β_5_FD). Partial correlations were also carried out between the median DC and GA at birth as a continuous variable (preterm and term-born neonates combined, controlling for PMA at scan, sex, FD outliers, and network density). Reported *P*-values were uncorrected, but false discovery rate procedure (Benjamini and Hochberg 1995) was applied to highlight *P*-values surviving multiple comparison correction.

The relationship between relative DC and outcome at 18 months was assessed both at the voxel and RSN levels. The relationships between voxel-wise DC and each outcome measure (Bayley-III subscales, CBCL subscales, and total Q-CHAT [all corrected for age at follow-up assessment]) were explored in GLMs, including PMA at scan, sex, network density, total number of FD outliers, tSNR, and IMD as covariates in the combined cohort of preterm- and term-born infants (*y* ~ β_0_ + β_1_PMA + β_2_Sex + β_3_Density + β_4_FD + β_5_Outcome Measure + β_6_IMD). To explore the relationship between DC and outcome at the RSN level, univariable analyses were conducted with partial correlations between median DC for each RSN and each outcome measure, controlling for PMA at scan, sex, network density, total number of FD outliers, IMD, and age at follow-up assessment as covariates. Multiple linear regression was also used to test if the median DC in the 11 RSNs significantly predicted outcome, again controlling for PMA at scan, sex, network density, total number of FD outliers, and IMD in the model. To assess whether the relationship between median DC and outcome differs with birth status (preterm vs. term), univariate analyses were also conducted in the term-born cohort and the preterm-born cohort separately, both at the voxel- and RSN level, and the interaction between median DC in each RSN and birth status was included in a multivariable model.

### Data availability

The dHCP is an open-access project. The imaging data used in this study were included in the 2021 (third) dHCP data release, which can be downloaded by completing a data usage agreement and by registering at http://data.developingconnectome.org.

## Results

### Functional centrality distribution in term-born neonates

**Fig. 1:**
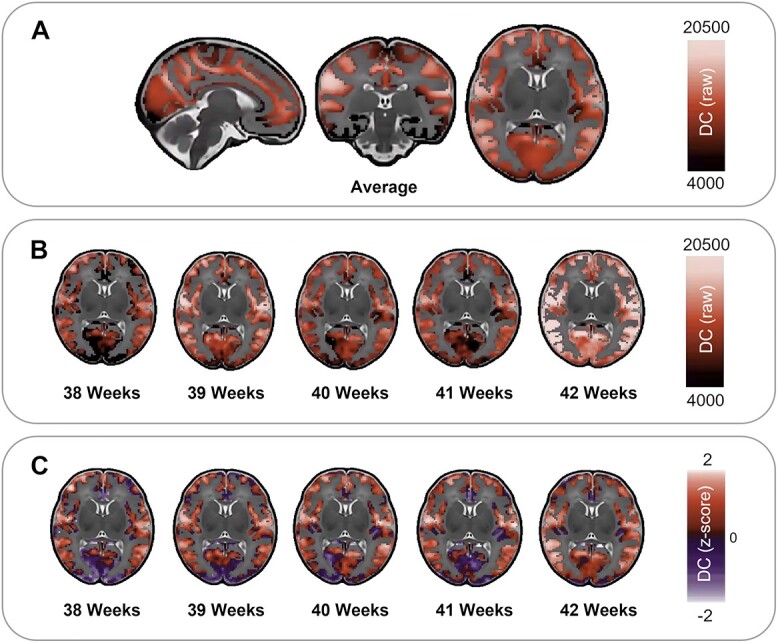
Average DC distribution in term-born neonates (A, raw values). Average DC raw values (B) and *z*-scored values (C) across postnatal weeks (PMA at scan) 38 to 42.

Analysis of the average brain connectivity matrices from term-born infants showed that the highest raw DC is evident within the left and right supramarginal gyri, the left and right precentral gyri, and bilaterally in the cuneus and is lowest in the inferior temporal regions (Fig. 1A). Panels B and C in Fig. 1 show the weekly average DC maps as raw values and *z*-scored values, respectively.

### Association of functional centrality with age in term-born neonates

**Fig. 2:**
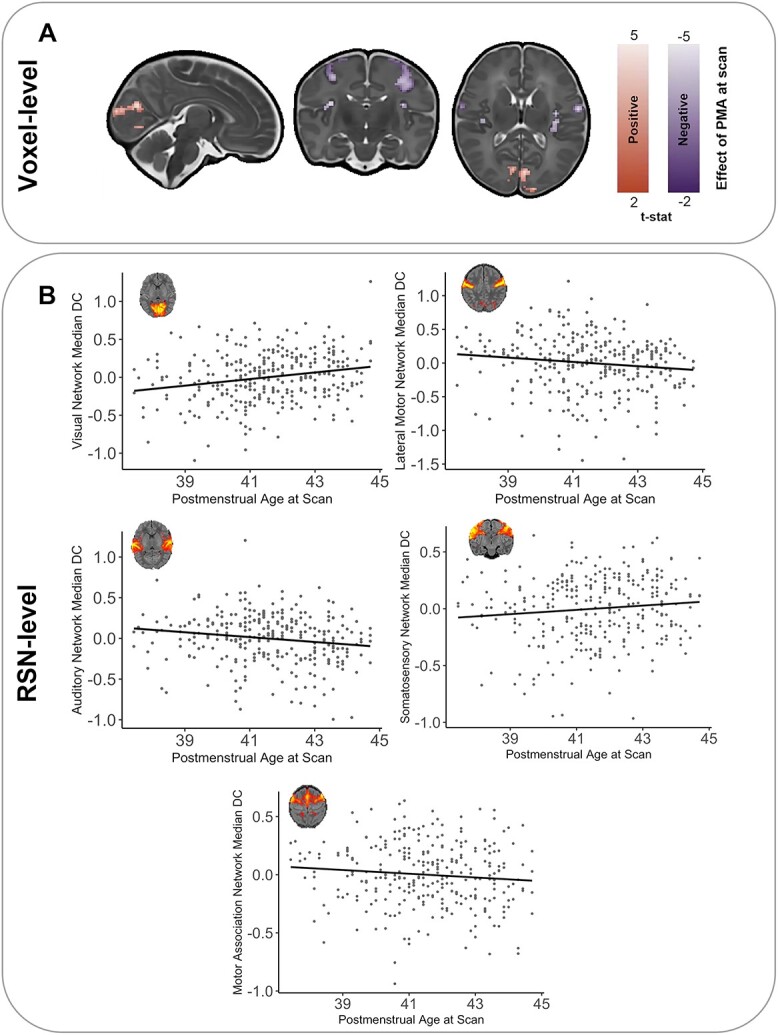
Effect of PMA at scan on DC in term-born infants. A) PMA at scan was associated with voxel-wise regional increases (red) and decreases (purple) in relative degree (T-statistic, *P* < 0.025). B) Significant relationships were also present between the relative median DC at the RSN-level and weeks of PMA at scan (*z*-score, residuals corrected for GA at birth, sex, FD outliers, and density). RSN figures adapted from Eyre et al. (2021).

In term-born infants, increasing PMA at scan was associated with an increase in voxel-wise relative DC within the cuneus and calcarine fissure and small regions within the lingual gyrus and superior occipital gyrus. PMA at scan was also associated with a decrease in relative DC in the bilateral precentral and postcentral gyrus in addition to the rolandic operculum, insula, and small regions within the transverse temporal gyrus, the right angular gyrus, and the right inferior parietal gyrus (corrected for PND, sex, FD motion outliers, network density, and tSNR, Fig. 2A).

A similar pattern of results was observed when exploring median DC in 11 established neonatal functional RSNs (Eyre et al. 2021). After correcting for multiple comparisons, there was a significant positive relationship between the median relative DC and PMA in the visual RSN (*r* = 0.30, *P* < 0.001). A negative relationship was also observed in the auditory RSN (*r* = −0.22, *P* < 0.001) and the lateral motor RSN (*r* = −0.17, *P* = 0.003, Fig. 2B). Correlations were also present within the somatosensory RSN and the motor association RSN (*r* = 0.15, *P* = 0.012 and *r* = −0.13, *P* = 0.021, respectively).

### Comparing functional centrality in term and preterm infants

**Fig. 3:**
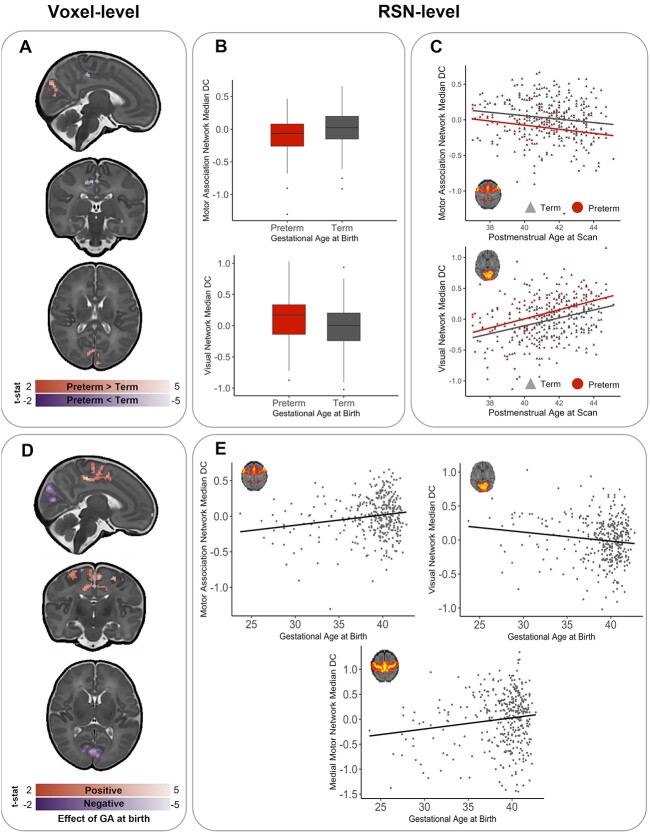
Effect of preterm birth on DC at term-equivalent age. A) Preterm-born infants show areas of voxel-wise regionally higher (red) and lower (blue) relative functional centrality compared to term-born infants. B) Significant differences were also present between preterm-born and term-born infants at the RSN level. C) There was no interaction between birth status (preterm-birth vs. term-birth) and PMA at scan (in weeks). D) When GA at birth was treated as a continuous variable (in weeks), it was associated with voxel-wise regional increases (red) and decreases (purple) in relative functional centrality (T-statistic, *P* < 0.025) at term-equivalent age. E) Significant relationship was also present between the relative median DC at the RSN-level and GA (Z = score, residuals, corrected for PMA at scan, sex, FD outliers, and density. Representative RSN figures adapted from Eyre et al. (2021).

At the voxel-level, preterm-born infants showed a higher relative DC in the right and left cunei and the left and right calcarine fissures. Higher DC was also observed in very limited regions within the left middle and inferior frontal gyri. Preterm-born infants showed lower centrality predominantly within the right cingulum and precunueus in addition to the left paracentral lobule and the right and left precentral gyrus (corrected for PMA at scan, sex, motion outliers, network density, and tSNR, Fig. 3A). For cluster sizes, see Supplementary Table 2.

When assessing the median DC within established neonatal functional RSNs, preterm-born infants showed a decreased median DC in the motor association RSN (*P* < 0.001) and increased centrality in the visual RSN (*P* = 0.009) when compared with term-born controls (Fig. 3B). There was no statistically significant interaction between group (term/preterm) and PMA at a voxel or RSN level (Fig. 3C).

In the combined cohort of term-born and preterm-born neonates at term-equivalent age, younger GA at birth resulted in lower voxel-wise relative DC in the bilateral supplementary motor areas, the paracentral lobules, and the right and left cingulum and precuneus in addition to the bilateral precentral and middle frontal gyri and a number of small clusters in areas listed in Supplementary Table 3. Younger GA at birth was also associated with an increased relative centrality in the right and left calcarine fissures and cunei in addition to the left superior occipital gyrus, regions within the frontal cortex, and a number of small clusters in areas listed in Supplementary Table 3 (corrected for PMA at scan, sex, motion outliers, network density, and tSNR, Fig. 3D).

At the RSN level, the median relative DC in the motor association RSN and medial motor RSN were positively related to GA at birth (*r* = 0.19, *P* < 0.001 and *r* = 0.15, *P* = 0.004, respectively), and median DC in the visual RSN was negatively associated with GA at birth (*r* = −0.15, *P* = 0.004), significant after multiple comparison correction (Fig. 3E).

### Effect of network density and tSNR

To understand the effect of network density and tSNR on neonatal DC, the effect of including these variables in analysis was explored. Inclusion of network density and tSNR as covariates in the voxel-wise analyses did not significantly alter the location of results but did impact on the magnitude (see Supplementary Figs. 1–3).

We also repeated the main voxel-wise statistical analyses on networks thresholded to include all connections (>0) and on networks thresholded to only include the strongest 10% of connections (10% proportional threshold). Our results were largely replicated (see Supplementary Figs. 4–6), suggesting that the observed effects are not dependent on the choice of network threshold.

### The relationship between DC and outcome at 18 months

There were no voxel-wise relationships between relative DC and neurodevelopmental outcomes at 18 months significant after multiple comparison correction in the term- and preterm-born infants combined, in the term-born infants only, or in the preterm-born infants only. There were also no relationships that survived multiple comparison correction between DC and outcome at 18 months at the RSN level. Importantly, this was also the case for the regions affected by preterm birth at term-equivalent age.

Prior to multiple comparison correction, at a significance threshold of *P* < 0.05, some significant relationships were present. As this may be informative for future studies, these results have been included in the Supplementary Material. Briefly, prior to multiple comparison correction in the univariate analysis, CBCL Externalizing was related to median DC in the visual association network (*r* = −0.155, *P* = 0.009), and CBCL “other” was associated with median DC in the auditory network (*r* = 0.124, *P* = 0.038) in the term- and preterm-born infants combined (Supplementary Table 3). In the term-born infants, Bayley Communication was associated with median DC in the posterior-parietal RSN (*r* = −0.135, *P* = 0.038), and CBCL Internalizing was associated with median DC in the somatosensory RSN (*r* = −0.135, *P* = 0.039, Supplementary Table 4). In the preterm-born infants, CBCL Internalizing was associated with median DC in the auditory RSN (*r* = 0.359, *P* = 0.018), Bayley Motor was associated with median DC in the prefrontal RSN (*r* = −0.335, *P* = 0.026), and CBCL Externalizing was associated with the Visual Association RSN (*r* = −0.303, *P* = 0.049, Supplementary Table 5).

In the multivariable models, the only significant predictor prior to multiple comparison correction was the visual association RSN in the CBCL Externalizing model (Supplementary Table 6). When the interaction between birth status (preterm vs. term) and each of the 11 RSN networks were included in the models, significant interactions were present between median DC in the lateral motor RSN and birth status (preterm vs. term), as well as the motor association RSN and birth status, when predicting CBCL Externalizing (Supplementary Table 7).

## Discussion

The aim of this study was to characterize postnatal functional network centrality development in term-born infants at the voxel level in addition to exploring how centrality may be altered by preterm birth. Thus, we here provide a description of a voxel-wise measure of functional centrality (DC) in the neonatal brain and explore how it is associated with age at scan in term-born infants and GA at birth in a cohort of term-born and preterm-born infants. In the early postnatal period of term-born infants, our results suggest that the typical maturational trajectory involves an increase in relative functional centrality predominantly within the visual cortex and a decrease in relative functional centrality within the primary motor and auditory cortices. Further, preterm birth appears to affect centrality largely in the same brain areas, although alterations in functional centrality associated with preterm birth in the motor cortex correspond to largely medial and association RSNs compared to the lateral motor RSN being associated with age at scan in term infants. Comparing median values corresponding to previously defined neonatal RSNs (Eyre et al. 2021), preterm-born infants showed an increased functional centrality in the visual RSN and decreased centrality in the motor RSN. No statistically significant relationship was observed between functional centrality and developmental outcomes at 18 months.

The changes in relative functional centrality associated with age at scan in term-born infants are located within primary RSNs. The maturation of RSNs shows a primary-to-higher-order ontogenetic sequence, with RSNs encompassing primary motor, visual, and auditory cortices already showing adult-like topology in term-born infants (Eyre et al. 2021). This is in line with neonates demonstrating visual processing capabilities albeit somewhat limited (Brémond-Gignac et al. 2011), and the capability for movement (Einspieler et al. 2008), and tactile and auditory processing (Ferronato et al. 2014; Maitre et al. 2020) perinatally. Thus, the main changes in relative functional centrality associated with age at scan are within the RSNs which are the most substantially developed in early life and are important for early interactions with the environment.

While spontaneous retinal activity allows the visual pathways to start to function early in the third trimester, following birth, there is a large increase in visual stimuli, inducing increased cerebral activity and maturational mechanisms (Dubois et al. 2020). The fact that we observe an increase in relative centrality within the visual system may reflect the establishment and maturation of the visual pathways (Siu and Murphy 2018). This is in line with previous seed-based connectivity studies suggesting a quantitative increase in the connectivity of the visual RSN during the first months after birth (Gao et al. 2015). Although mapping the exact processes driving the increase in DC within the visual system is beyond the scope of the current study, we know that several visual perceptual functions are improving during this time. As reviewed by Siu and Murphy (2018), this includes initial steps in the development of spatial, contrast sensitivity, orientation sensitivity motion perception, color perception, contour integration, and face perception.

Similar to the visual network, the primary motor and auditory networks show adult-like topology at birth (Keunen et al. 2017). In contrast to the visual RSN however, we observed a decrease in relative functional centrality associated with age at scan within these RSNs. This may reflect an experience-expectant process whereby broad cortical circuits become increasingly refined with exposure, resulting in the postnatal specialization of connectivity. However, it is worth noting that previous work looking specifically at speech processing (Shultz et al. 2014) and auditory stimuli more generally (Gao et al. 2015) suggests that this process takes place slightly later than the age of infants included in the present study, warranting a more fine-grained assessment of the temporal trajectories of specialization within these RSNs to assess whether the changes we observe reflect refinement or a relative increase in the importance of other RSNs.

With the brain undergoing dramatic changes in functional activity during the third trimester, infants born prematurely during this sensitive period are vulnerable to the influence of developmentally unexpected sensory stimuli which is not age-appropriate and significantly different from the womb in duration, complexity, and intensity (Beltrán et al. 2021; Schmidt Mellado et.al. 2022). Such an exposure to inadequate and/or inappropriate visual, auditory, and motor stimuli may contribute to subtle brain changes both in primary cortices and associative areas, which can in turn translate into long-lasting sensorimotor alterations (El-Metwally and Medina 2020). Indeed, children who are born prematurely more often present with conditions related to disrupted sensory processing, such as ADHD and autism and learning and memory problems (El-Metwally and Medina 2020) in addition to neuromotor abnormalities (Wheelock et al. 2018).

Notably, the areas that appear to be most affected by preterm birth in the present study are also predominantly located within the motor and visual RSNs. This suggests that preterm birth affects the regions which are showing the most substantial development during the perinatal period. Given the crucial role of sensory input in neurodevelopment (Schmidt Mellado et.al. (2022), preterm infants exposed to developmentally unexpected stimulation of motor and visual networks (i.e. ex utero vs. in utero experience) may show an accelerated maturation along normal developmental trajectories or may adopt adverse and/or compensatory mechanisms as an adaptive response to cope with the environment (De Asis-Cruz et al. 2020; Eyre et al. 2021; Schmidt Mellado et.al. 2022). This may in turn result in the altered patterns of functional centrality which we observe in preterm-born infants in the current study. However, it is challenging to disentangle whether the alterations in functional centrality observed in preterm-born infants are a result of less time spent in utero and the consequent premature exposure to the postnatal environment or underlying clinical and/or genetic factors associated with preterm-birth.

As the preterm-born infants included in this study are predominantly moderate or late preterm, it is noteworthy that the differences in functional architecture are present even in this cohort. This evidences the importance of studying preterm-birth in a wide range of GAs at birth and not solely in infants born extremely preterm, particularly as 80% of preterm infants are born moderate or late preterm (Blencowe et al. 2012). It is, however, also worth noting that the present study population is a cohort of “healthy” preterm-born infants, with no incidental findings of likely clinical significance, who did not demonstrate significantly different developmental outcomes from term-born infants at 18 months. We are therefore unable to extrapolate our findings to more severely affected preterm babies, such as those with significant white matter damage.

The fact that we did not observe any robust relationships between functional centrality and outcome at 18 months that survived multiple comparison correction is in line with the majority of neonatal fMRI studies, where clear relationships between functional connectivity patterns in the perinatal period and outcome at 1–2 years of age are seldom reported, particularly in term-born cohorts. Detecting links between brain imaging and general measures of behavior is difficult in the developing brain, as the changes associated with normal maturation are much larger than those associated with individual differences (Batalle et al. 2018). The functional differences between individuals may be so subtle that they do not manifest behaviorally this early on, or the relationships may be more complex and nonlinear. There is also the possibility that subtle differences may be influenced or magnified by the ongoing environmental exposures throughout development (Livingston and Happé 2017) and that small functional differences in primary networks could disrupt or unbalance the developmental trajectories of higher-order networks (Ciarrusta et al. 2020), leading to behavioral manifestations later in development. Additionally, it is conceivable that the relationship between functional alterations associated with preterm birth and outcome might have been clearer in a cohort of extremely preterm infants and/or infants with more severe damage as a result of preterm birth. Indeed, previous studies have shown relationships between functional connectivity at term-equivalent age and outcome at 1–2 years of age in very preterm infants (Toulmin et al. 2021). It may also be the case that disruption to functional centrality topology observed at term-equivalent age in preterm-born infants may be “normalized” or be compensated for over the first year(s) of life so that early disruption does not result in behavioral consequences at 18 months.

Our study advances on previous work by providing a description of functional centrality in a large, representative sample. Our results offer complementary information to the findings by Cao et al. (2017), who demonstrated increases in absolute DC predominantly within the precentral and postcentral gyri and supplementary motor area and decreases within the posterior cingulate and precuneus cortex from 31 to 42 weeks PMA. The current findings provide a picture of the alterations in relative centrality which are associated with preterm birth at term-equivalent age. Although our results show somewhat different patterns of altered centrality associated with preterm birth than those reported by Gozdas et al. (2018), a direct comparison is not possible due to differences in methodology and sample—most notably, their comparison between groups being carried out on a regional basis using 90 ROIs rather than a voxel-based approach.

Our study has several strengths. We use a large cohort and employ a metric that does not rely on a particular parcellation template or selection of a priori ROIs. The imaging system for the dHCP is optimized for neonatal imaging. The close-fitting custom head coil provides exceptional SNR on the cortical surface (Hughes et al. 2017). However, this does result in a bias toward surface-proximate sources, which is further compounded by the use of highly accelerated multiband EPI (Fitzgibbon et al. 2020). Because of this, we did not include deep gray matter in our analysis, including regions such as the amygdala, thalamus, and cerebellum. Additionally, despite the dHCP functional pipeline including advanced distortion correction techniques (Fitzgibbon et al. 2020), some signal is lost due to the air/tissue and bone/tissue interfaces. The use of a single phase-encode direction (anterior–posterior) may also compress signal in the frontal regions. To mitigate these effects, voxel-wise tSNR was included as a covariate in voxel-wise analyses. As motion is known to have an impact on the rs-fMRI signal (Power et al. 2012; Satterthwaite et al. 2012) and the term-born infants presented with more motion than the preterm-born infants, we cannot exclude the possibility that motion artifacts may have had an impact on our results. However, the dHCP functional pipeline includes multiple steps to mitigate the effect of motion and physiological confounds to minimize data loss; in addition, we excluded babies with high motion and included number of FD outliers as a covariate in analyses. These steps should also have helped to mitigate the effect of differences in sleep state and arousal as these manifest in different levels of subject motion (Gu et al. 2020; Prechtl 1974). We are, however, unable to account for any fundamental differences in the BOLD signal in term-born and preterm-born infants linked to cerebrovascular factors (Bouyssi-Kobar et al. 2018). Additionally, while acknowledging that the role of network density in graph theory-based analyses is a complex issue (van den Heuvel et al. 2017; Hallquist and Hillary 2019), we are able to make some inferences about the importance of network density in our analyses. The inclusion of density as a covariate did have an effect on magnitude of our findings, but did not change their location, suggesting that the observed regional differences are not solely due to differences in global network topology. To further establish to what degree our results were dependent on the density of the analyzed networks, we also conducted our main analyses at 2 additional network thresholds. There is currently no consensus on the most advantageous thresholding approaches to use in voxel-based brain network analyses. On the one hand, the argument can be made for assessing sparser networks with potentially fewer spurious connections and more physiologically plausible densities (Zalesky et al. 2016). However, it has also been argued that the removal of weak connections in dense connectomes is not necessarily beneficial and a threshold-free approach is more appropriate (Civier et al. 2019). We therefore replicated our analyses with networks, including all positive connections (>0), and networks thresholded at a proportional threshold of 10% (i.e. including only the strongest 10% of connections). These additional analyses mostly replicated our main findings using an absolute threshold of 0.2, supporting the robustness of the results reported.

Our study is also limited by the fact that our outcome assessments are only available at 18 months of age, meaning we are unable to establish whether the patterns of atypical functional connectivity we observe to be associated with preterm birth translate into behavioral or socio-emotional difficulties commonly observed in preterm-born infants in later life, or whether they reflect a transient pattern of altered functional architecture. Further work is needed to understand whether DC at birth holds any predictive value for behavioral outcomes at later timepoints. Lastly, although DC is a widely used method to study functional network centrality, various other centrality measures exist and may capture other aspects of functional connectivity (Zuo et al. 2012).

In summary, this work contributes to our understanding of typical maturational trajectories of functional centrality at the voxel and RSN levels and explores how functional centrality is affected by preterm birth. Our findings suggest that the changes in centrality associated with typical maturation and preterm birth are both predominantly located within the primary motor and visual cortices. This suggests that preterm birth largely affects the pattern of functional centrality of the cortical regions that undergo the most substantial development in the perinatal period, highlighting both the sensitive and significant natures of these regions during early life. As functional centrality was not related to performance on outcome measures at 18 months, further work is needed to ascertain whether the alterations in functional centrality associated with preterm birth are adaptive and serve a compensatory role, or whether they are disruptive and potentially predictive of neurodevelopmental features that are more subtle than those measured here at 18 months, or that emerge later in life.

## Supplementary Material

DC_Manuscript_SupplementaryMaterial_Final_bhac444
