# Supplementary material for: Development of neonatal brain functional centrality and alterations associated with preterm birth
Source: Cereb Cortex. 2022 Nov 20;33(9):5585–96. doi: 10.1093/cercor/bhac444 (PMC10152096; doi:10.1093/cercor/bhac444)
Supplement: DC_Manuscript_SupplementaryMaterial_Final_bhac444 [file dc_manuscript_supplementarymaterial_final_bhac444.zip › DC_Manuscript_SupplementaryMaterial_Final_bhac444.pdf]

# Development of neonatal brain functional centrality and alterations associated with preterm birth

Sunniva Fenn-Moltu[a,b], Sean P Fitzgibbon[c], Judit Ciarrusta[a,b], Michael Eyre[b], Lucilio Cordero-Grande[b,d], Andrew Chew[b], Shona Falconer[b], Oliver Gale-Grant[b,e] , Nicholas Harper[b], Ralica Dimitrova[b], Katy Vecchiato[a,b], Daphna Fenchel[a,b,e], Ayesha Javed[a,b], Megan Earl[a,b,f], Anthony N Price[b], Emer Hughes[b], Eugene P Duff[c,g], Jonathan O'Muircheartaigh[a,b,e], Chiara Nosarti[b,h], Tomoki Arichi[b,e,i,j], Daniel Rueckert[k,l], Serena Counsell[b], Joseph V Hajnal[b], A David Edwards[b,e]*, Grainne McAlonan[a,e]*, Dafnis Batalle[a,b]*†,

a) Department of Forensic and Neurodevelopmental Sciences, Institute of Psychiatry, Psychology & Neuroscience, King's College London, London, UK
b) Centre for the Developing Brain, School of Biomedical Engineering & Imaging Sciences, King's College London, London, UK
c) Wellcome Centre for Integrative Neuroimaging (WIN FMRIB), University of Oxford, Oxford, UK
d) Biomedical Image Technologies, ETSI Telecomunicación, Universidad Politécnica de Madrid & CIBER-BBN, Madrid, Spain
e) MRC Centre for Neurodevelopmental Disorders, King's College London
f) Paediatric Liver GI and Nutrition Centre and MowatLabs, King's College London
g) Department of Paediatrics, University of Oxford, Oxford, UK
h) Department of Child and Adolescent Psychiatry, Institute of Psychiatry Psychology and Neuroscience, King's College London, London, United Kingdom
i) Paediatric Neurosciences, Evelina London Children's Hospital, Guy's and St Thomas' NHS Foundation Trust, United Kingdom
j) Department of Bioengineering, Imperial College London, United Kingdom
k) Biomedical Image Analysis Group, Imperial College London, London, UK
l) Institute for AI and Informatics in Medicine, Klinikum rechts der Isar, Technical University of Munich, Germany

*Joint senior authors

†Corresponding author:

Dr. Dafnis Batalle
Department of Forensic and Neurodevelopmental Science
Institute of Psychiatry, Psychology & Neuroscience
King's College London 16, De Crespigny Park, SE5 8AF, London, UK
+44-(0)-207-848-0922 | dafnis.batalle@kcl.ac.uk

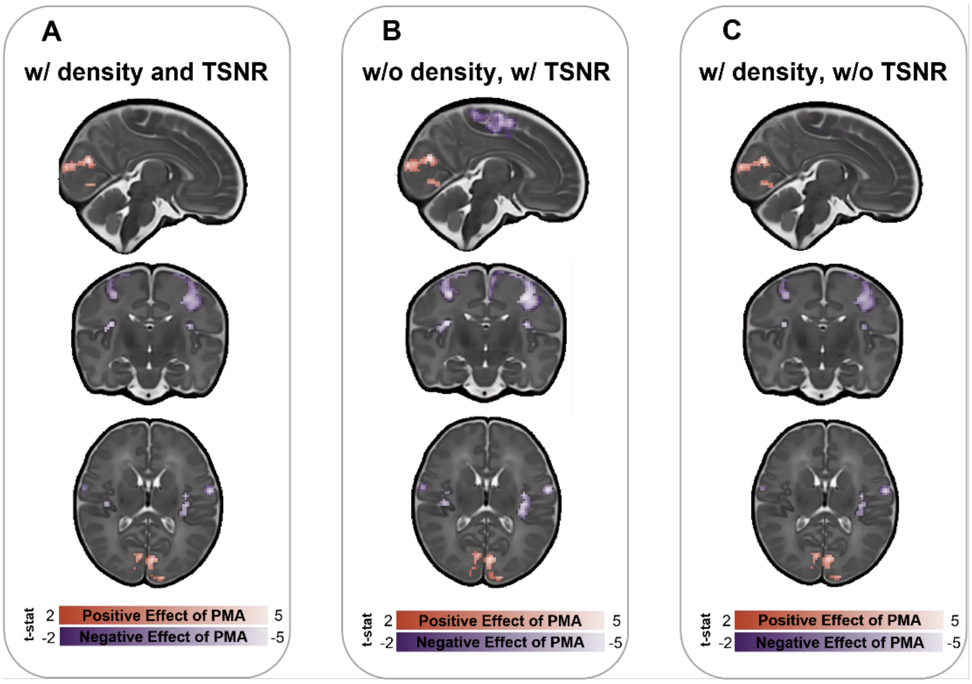


**Supplementary Figure 1.** Postmenstrual age at scan, including density and tSNR as covariates **(A),** including tSNR but not density **(B),** and including density but not tSNR **(C).**

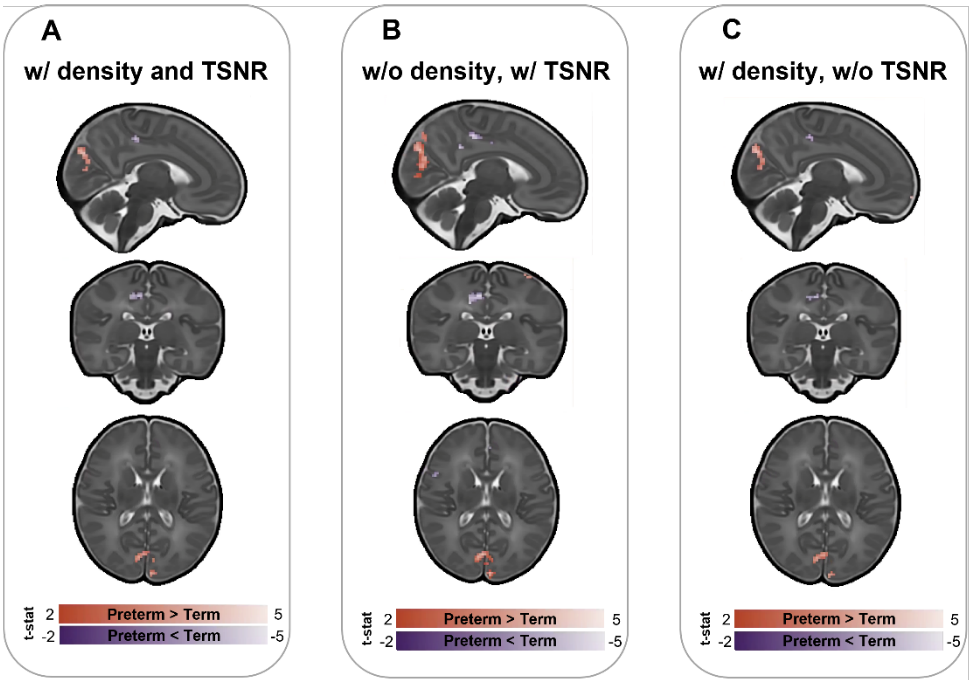


**Supplementary Figure 2.** Preterm versus term, including density and tSNR as covariates **(A),** including tSNR but not density **(B),** and including density but not tSNR **(C).**

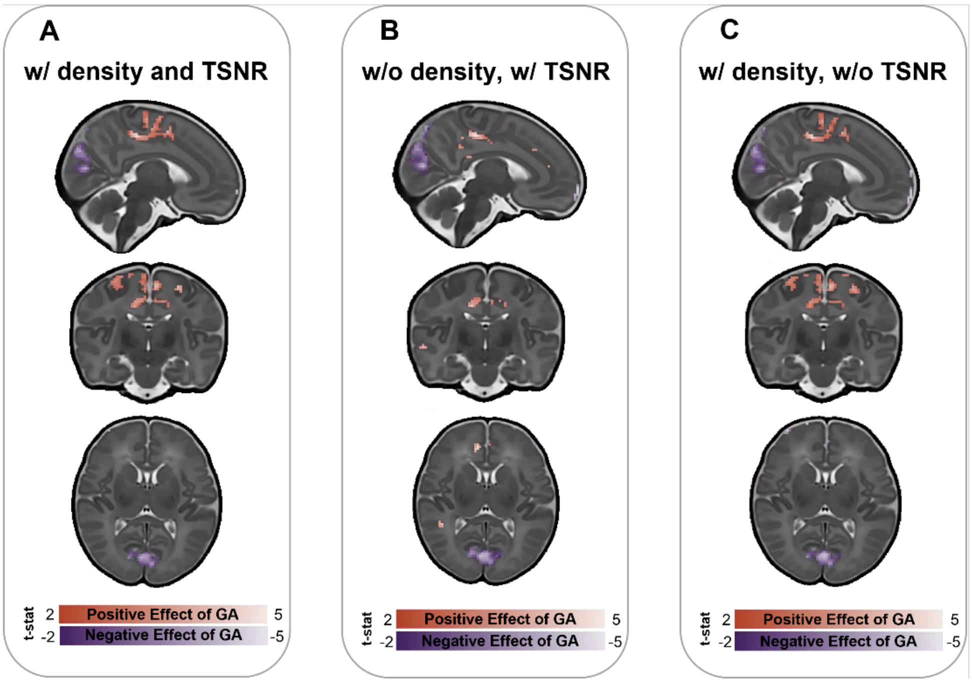


**Supplementary Figure 3.** Gestational age at birth, including density and tSNR as covariates (**A**), including tSNR but not density **(B)**, and including density but not tSNR **(C).**

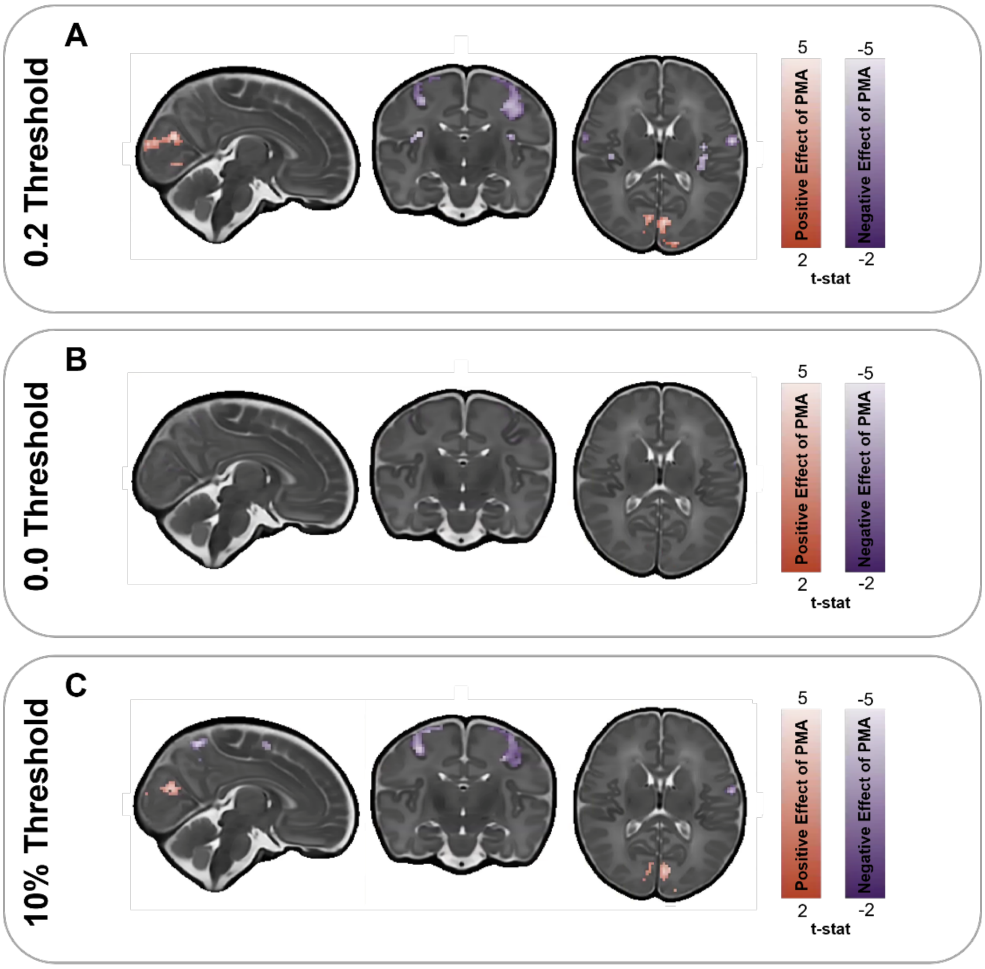

**Supplementary Figure 4.** Voxel-wise association of DC with postmenstrual age at scan with networks thresholded with an absolute threshold of 0.2 **(A),** >0.0 **(B)** and 10% proportional threshold **(C).**

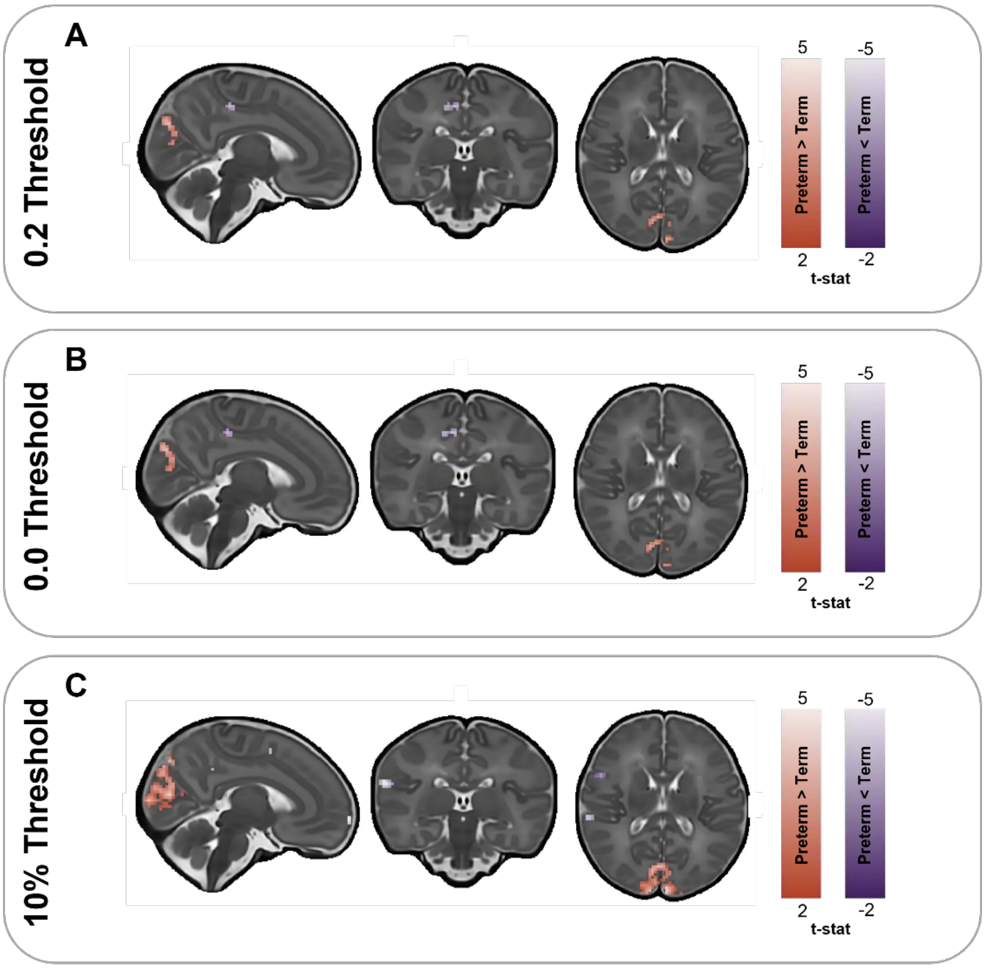


**Supplementary Figure 5.** Voxel-wise association of differences in DC between term-born and preterm-born neonates with networks thresholded with an absolute threshold of 0.2 **(A),** >0.0 **(B)** and 10% proportional threshold **(C).**

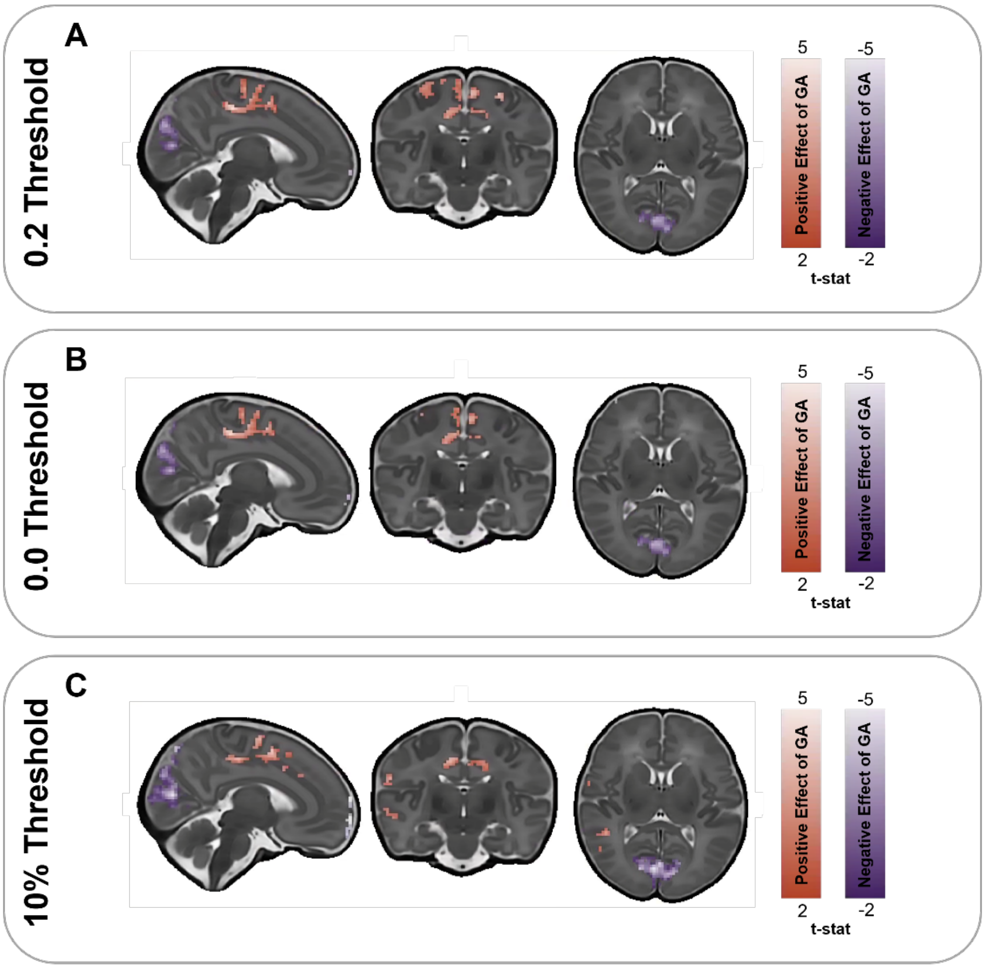


**Supplementary Figure 6.** Voxel-wise association of DC with gestational age at birth with networks thresholded with an absolute threshold of 0.2 **(A),** >0.0 **(B)** and 10% proportional threshold **(C).**
**(C).**

## Supplementary Table 1.

Association of functional centrality with PMA at scan in Term-Born Neonates - Clusters (>10 voxels)

| Cluster | Size (voxels) |
|---|---|
| ***Clusters Showing Increased DC with PMA*** | |
| Cuneus_L, Occipical_Sup_L, Calcarine_L | 133 |
| Occipital_Sup_R | 49 |
| Cuneus_R, Calcarine_R | 29 |
| Lingual_R | 19 |
| Lingual_L | 10 |
| Cuneus_L | 10 |
| ***Clusters Showing Decreased DC with PMA*** | |
| Precentral_L, Postcentral_L | 754 |
| Precentral_R, Postcentra_R | 483 |
| Rolandic_Oper_L, Insula_L, Heschl_L | 67 |
| Rolandic_Oper_R, Insula_R, Heschl_R. | 40 |
| Parietal_Inf_R, Angular R | 12 |

## Supplementary Table 2.

Effect of Preterm birth on degree centrality at Term-Equivalent Age – Group-wise analysis - Clusters (>10 voxels)

| Cluster | Size (voxels) |
|---|---|
| ***Clusters Showing Higher DC in Preterm compared to Term-born neonates*** | |
| Calcarine_L,Calcarine_R, Cuneus_L,Cuneus_R | 261 |
| Frontal_Inf_Tri_L, Frontal_Mid_L,Frontal_Mid_Orb_L | 23 |
| ***Clusters Showing Lower DC in Preterm compared to Term-born neonates*** | |
| Cluster 4 Cingulum_Mid_R, Precuneus_R | 43 |
| Precentral_R | 10 |
| Cluster 2 Precentral_L, Paracentral_Lobule_L | 10 |

## Supplementary Table 3.

Effect of Preterm birth on degree centrality at Term-Equivalent Age – Continuous GA - Clusters (>10 voxels)

| Cluster | Size (voxels) |
|---|---|
| ***Clusters Showing Increasing DC with GA*** | |
| Supp_Motor_Area_R, Cingulum_Mid_R, Paracentral_Lobule_R, Precuneus_R | 382 |
| Supp_Motor_Area_L,Cingulum_Mid_L, Paracentral_Lobule_L, Precuneus_L | 200 |
| Precentral_R, Frontal_Mid_R | 83 |
| Precentral_L, Frontal_Mid_L | 40 |
| ParaHippocampal_L, | 39 |
| Frontal_Inf_Oper_R, Rolandic_Oper_R, Insula_R | 21 |
| Rolandic_Oper_R | 18 |
| Precentral_L,Supp_Motor_Area_L,Frontal_Sup_L | 17 |
| Paracentral_Lobule_R,Precentral_R, Postcentral_R | 14 |
| Precuneus_L,Cingulum_Mid_R,Cingulum_Mid_L | 12 |
| ***Clusters Showing Decreasing DC with GA*** | |
| Cuneus_L, Cunues_R, Calcarine_L, Calcarine_R, Occipital_Sup_L | 570 |
| Frontal_Inf_Tri_R,Frontal_Inf_Orb_R, Frontal_Mid_R, Frontal_Mid_Ord_R | 89 |
| Frontal_Sup_R, Frontal_Sup_Medial_R, Frontal_Sup_Orb_R,Frontal_Med_Ord_R | 24 |
| Occipital_Mid_L,Angular_L | 13 |
| Frontal_Inf_Tri_L, Frontal_Mid_L | 12 |
| Parietal_Sup_R | 12 |

## Supplementary Table 4.

***Univariable Analyses – term & preterm***

| | Medial Motor | Lateral Motor | Somato-sensory | Motor Association | Auditory | Visual | Tempero-parietal | Posterior-parietal | Fronto-parietal | Prefrontal | Visual Association |
|---|---|---|---|---|---|---|---|---|---|---|---|
| **Bayley Cognition** | r=-0.0514 (p=0.387) | r=-0.0636 (p=0.284) | r=0.0303 (p=0.609) | r=-0.022 (p=0.711) | r=-0.0907 (p=0.126) | r=-0.00948 (p=0.873) | r=-0.0218 (p=0.714) | r=0.0258 (p=0.713) | r=0.0495 (p=0.404) | r=0.0609 (p=0.305) | r=-0.032 (p=0.590) |
| **Bayley Communication** | r=0.0366 (p=0.538) | r=-0.0378 (p=0.525) | r=0.0585 (p=0.325) | r=-0.00792 (p=0.894) | r=-0.0781 (p=0.188) | r=0.0224 (p=0.706) | r=-0.0279 (p=0.639) | r=0.102 (p=0.0896) | r=0.052 (p=0.342) | r=-0.0066 (p=0.901) | r=-0.0224 (p=0.689) |
| **Bayley Motor** | r=-0.0595 (p=0.316) | r=-0.0479 (p=0.420) | r=0.0432 (p=0.466) | r=-0.0347 (p=0.559) | r=-0.0753 (p=0.204) | r=0.0073 (p=0.902) | r=-0.0246 (p=0.679) | r=0.0546 (p=0.358) | r=0.077 (p=0.194) | r=0.0121 (p=0.838) | r=-0.0191 (p=0.748) |
| **CBCL Externalising** | r=0.039 (p=0.515) | r=-0.019 (p=0.748) | r=-0.0296 (p=0.622) | r=0.0237 (p=0.692) | r=0.0481 (p=0.422) | r=0.0275 (p=0.646) | r=-0.0279 (p=0.641) | r=-0.0678 (p=0.257) | r=-0.0209 (p=0.728) | r=0.0732 (p=0.221) | **r=-0.155 (p=0.00943)** |
| **CBCL Internalising** | r=-0.0234 (p=0.696) | r=-0.0106 (p=0.859) | r=-0.0903 (p=0.131) | r=-0.0474 (p=0.429) | r=0.0574 (p=0.338) | r=0.101 (p=0.0909) | r=0.00203 (p=0.973) | r=-0.0697 (p=0.244) | r=-0.0425 (p=0.477) | r=0.0218 (p=0.716) | r=-0.0394 (p=0.511) |

| | | | | | | | | | | | |
|---|---|---|---|---|---|---|---|---|---|---|---|
| **CBCL *Other*** | r=0.0104 (p=0.862) | r=0.0588 (p=0.326) | r=-0.0412 (p=0.491) | r=-0.0479 (p=0.423) | **r=0.124 (p=0.0378)** | r=0.0658 (p=0.271) | r=-0.0525 (p=0.381) | r=-0.0572 (p=0.340) | r=-0.065 (p=0.277) | r=0.0142 (p=0.813) | r=-0.0101 (p=0.0913) |
| **QCHAT** | r=0.000707 (p=0.991) | r=-0.019 (p=0.751) | r=-0.0542 (p=0.366) | r=-0.036 (p=0.548) | r=0.0425 (p=0.478) | r=0.0489 (p=0.414) | r=0.0323 (p=0.590) | r=-0.0678 (p=0.257) | r=-0.0861 (p=0.150) | r=-0.0265 (p=0.658) | r=-0.0059 (p=0.922) |

Partial correlations between each median degree centrality in each RSN and each outcome measure at 18 months, controlling for IMD, PMA at scan, sex, total number of FD outliers, and network density in the combined cohort of term-born (n=300) and preterm-born (n=66) infants.

## Supplementary Table 5.

***Univariable Analyses – term only***

| | **Medial Motor** | **Lateral Motor** | **Somato-sensory** | **Motor Association** | **Auditory** | **Visual** | **Tempero-parietal** | **Posterior-parietal** | **Fronto-parietal** | **Prefrontal** | **Visual Association** |
|---|---|---|---|---|---|---|---|---|---|---|---|
| **Bayley Cognition** | r=0.0425 (p=0.515) | r=-0.078 (p=0.233) | r=-0.0255 (p=0.696) | r=-0.0515 (p=0.431) | r=-0.0792 (p=0.226) | r=-0.0133 (p=0.839) | r=-0.0366 (p=0.576) | r=0.051 (p=0.436) | r=0.0498 (p=0.447) | r=0.0387 (p=0.554) | r=-0.0319 (p=0.626) |
| **Bayley Communication** | r=0.0369 (p=0.573) | r=-0.0724 (p=0.268) | r=0.041 (p=0.531) | r=-0.064 (p=0.327) | r=-0.0944 (p=0.148) | r=-0.0699 (p=0.285) | r=-0.0447 (p=0.494) | **r=-0.135 (p=0.0384)** | r=0.06 (p=0.359) | r=-0.0195 (p=0.766) | r=-0.0181 (p=0.782) |
| **Bayley Motor** | r=0.072 (p=0.271) | r=-0.0654 (p=0.317) | r=0.0594 (p=0.364) | r=0.00209 (p=0.974) | r=-0.0598 (p=0.360) | r=0.019 (p=0.771) | r=-0.0293 (p=0.655) | r=-0.0646 (p=0.323) | r=0.0951 (p=0.145) | r=-0.0156 (p=0.812) | r=0.000894 (p=0.989) |
| **CBCL Externalising** | r=0.0639 (p=0.332) | r=0.0332 (p=0.614) | r=-0.0579 (p=0.379) | r=-0.052 (p=0.429) | r=0.0601 (p=0.361) | r=0.0304 (p=0.644) | r=0.00655 (p=0.921) | r=-0.0424 (p=0.519) | r=-0.0441 (p=0.503) | r=0.0323 (p=0.623) | r=-0.116 (p=0.077) |
| **CBCL Internalising** | r=0.007 (p=0.915) | r=-0.0211 (p=0.749) | **r=-0.135 (p=0.0391)** | r=-0.0777 (p=0.237) | r=0.0189 (p=0.774) | r=0.119 (p=0.0691) | r=-0.00845 (p=0.898) | r=-0.076 (p=0.248) | r=-0.0567 (p=0.389) | r=-0.00247 (p=0.97) | r=-0.0119 (p=0.857) |
| **CBCL *Other*** | r=-0.0612 (p=0.352) | r=0.0823 (p=0.211) | r=-0.0654 (p=0.320) | r=0.0139 (p=0.211) | r=0.114 (p=0.0828) | r=0.0673 (p=0.306) | r=-0.0361 (p=0.584) | r=-0.0407 (p=0.536) | r=-0.0739 (p=0.261) | r=-0.0378 (p=0.565) | r=-0.0795 (p=0.227) |
| **QCHAT** | r=0.0363 (p=0.582) | r=0.017 (p=0.796) | r=-0.065 (p=0.323) | r=-0.043 (p=0.514) | r=0.0173 (p=0.793) | r=0.0268 (p=0.684) | r=0.045 (p=0.494) | r=-0.0764 (p=0.245) | r=-0.082 (p=0.212) | r=-0.0218 (p=0.740) | r=-0.0149 (p=0.821) |

Partial correlations between each median degree centrality in each RSN and each outcome measure at 18 months, controlling for IMD, PMA at scan, sex, total number of FD outliers, and network density in the term-born (n=300) infants.

## Supplementary Table 6.

***Univariable Analyses –preterm only***

| | **Medial Motor** | **Lateral Motor** | **Somato-sensory** | **Motor Association** | **Auditory** | **Visual** | **Tempero-parietal** | **Posterior-parietal** | **Fronto-parietal** | **Prefrontal** | **Visual Association** |
|---|---|---|---|---|---|---|---|---|---|---|---|
| **Bayley Cognition** | r=0.0629 (p=0.681) | r=-0.0818 (p=0.593) | r=-0.00019 (p=0.999) | r=0.115 (p=0.453) | r=-0.148 (p=0.333) | r=-0.198 (p=0.191) | r=-0.0398 (p=0.795) | r=-0.158 (p=0.301) | r=-0.0695 (p=0.650) | r=0.246 (p=0.104) | r=-0.0562 (p=0.714) |
| **Bayley Communication** | r=-0.168 (p=0.277) | r=0.0731 (p=0.637) | r=0.0488 (p=0.753) | r=0.147 (p=0.343) | r=-0.112 (p=0.470) | r=-0.118 (p=0.446) | r=-0.0366 (p=0.814) | r=-0.0283 (p=0.855) | r=0.0495 (p=0.749) | r=-0.229 (p=0.135) | r=0.0269 (p=0.863) |
| **Bayley Motor** | r=-0.181 (p=0.239) | r=-0.009 (p=0.954) | r=-0.0626 (p=0.686) | r=-0.198 (p=0.198) | r=-0.214 (p=0.163) | r=-0.0662 (p=0.669) | r=-0.113 (p=0.467) | r=-0.163 (p=0.291) | r=-0.114 (p=0.461) | **r=-0.335 (p=0.0264)** | r=-0.135 (p=0.383) |
| **CBCL Externalising** | r=-0.104 (p=0.506) | r=-0.290 (p=0.0589) | r=0.0153 (p=0.923) | r=0.272 (p=0.0778) | r=-0.0169 (p=0.914) | r=0.0159 (p=0.919) | r=-0.190 (p=0.222) | r=-0.194 (p=0.213) | r=-0.00382 (p=0.981) | r=0.242 (p=0.118) | **r=-0.303 (p=0.0485)** |
| **CBCL Internalising** | r=-0.106 (p=0.498) | r=0.0609 (p=0.698) | r=0.115 (p=0.463) | r=0.0625 (p=0.690) | **r=0.359 (p=0.0181)** | r=-0.0663 (p=0.673) | r=-0.00357 (p=0.982) | r=-0.180 (p=0.249) | r=-0.173 (p=0.266) | r=0.0834 (p=0.595) | r=-0.219 (p=0.159) |
| **CBCL Other** | r=-0.134 (p=0.393) | r=-0.115 (p=0.464) | r=0.0966 (p=0.538) | r=0.157 (p=0.315) | r=0.222 (p=0.153) | r=0.0366 (p=0.816) | r=-0.164 (p=0.292) | r=-0.222 (p=0.153) | r=-0.129 (p=0.411) | r=0.119 (p=0.447) | r=-0.224 (p=0.149) |
| **QCHAT** | r=-0.0965 (p=0.538) | r=-0.143 (p=0.361) | r=0.0173 (p=0.912) | r=-0.267 (p=0.083) | r=0.223 (p=0.151) | r=0.143 (p=0.361) | r=0.015 (p=0.924) | r=-0.0126 (p=0.936) | r=-0.0577 (p=0.713) | r=-0.0769 (p=0.624) | r=-0.0346 (p=0.826) |

Partial correlations between each median degree centrality in each RSN and each outcome measure at 18 months, controlling for IMD, PMA at scan, sex, total number of FD outliers, and network density in the preterm-born (n=66) infants.

## Supplementary Table7

***Multivariable Analyses – w/o interaction terms***

| **Outcome Variable** | **Overall Model** | **Significant RSN predictors** |
|---|---|---|
| **Bayley Cognition** | **F(17,273)=2.075, p=0.008, multiple $R^2$ = 0.1144, adjusted $R^2$ = 0.0593** | |
| **Bayley Communication** | **F(17,273)=1.906, p=0.018, multiple $R^2$ = 0.1061, adjusted $R^2$ = 0.0504** | |
| Bayley Motor | F(17,273)=1.474, p=0.1035 (NS), multiple $R^2$ = 0.0841, adjusted $R^2$ = 0.0270 | |
| **CBCL Externalising** | **F(17,268)=1.725, p=0.039, multiple $R^2$ = 0.0986, adjusted $R^2$ = 0.0415** | **Visual Association Network (*b*=-0.275, t=-2.562, p=0.011)** |
| CBCL Internalising | F(17,268)=1.314, p=0.183 (NS), multiple $R^2$ = 0.077, adjusted $R^2$ = 0.0184 | |
| CBCL Other | F(17,268)=1.284, p=0.202 (NS), multiple $R^2$ = 0.075, adjusted $R^2$ = 0.0167 | |
| QCHAT | F(17,269)=1.353, p=0.160 (NS), multiple $R^2$ = 0.079, adjusted $R^2$ = 0.0206 | |

Multiple linear regressions to predict each outcome measure, given the 11 RSNs, PMA at scan, sex, total number of FD outliers and network density in the combined cohort of term-born (n=300) and preterm-born (n=66) infants.

# Supplementary Table 8.

***Multivariable Analyses – w/ interaction terms***

| Outcome Variable | Overall Model | Significant RSN predictors |
|---|---|---|
| Bayley Cognition | F(28,262)=1.147, p=0.2844 (NS), multiple $R^2$ = 0.1092, adjusted $R^2$ = 0.01396 | |
| Bayley Communication | F(28,262)=1.378, p=0.1034 (NS), multiple $R^2$ = 0.1284, adjusted $R^2$ = 0.0352 | |
| Bayley Motor | F(28,262)=0.9908, p=0.4829 (NS), multiple $R^2$ = 0.0957, adjusted $R^2$ = 0.0009 | |
| **CBCL Externalising** | **F(28,257)=1.742, p=0.01413, multiple $R^2$ = 0.1595, adjusted $R^2$ = 0.06797** | **Lateral Motor (*b*=-0.5048, t=-2.510, p=0.01268), Motor Association (*b*=0.3302, t=-2.016, p=0.0448), Lateral Motor*Birth Status (*b*=0.4836, t=-2.198, p=0.02885), Motor Association*Birth Status (*b*=-0.5015, t=-2.899, p=0.00407)** |
| CBCL Internalising | F(28,257)=1.192, p=0.2378 (NS), multiple $R^2$ = 0.115, adjusted $R^2$ = 0.01854 | |
| CBCL Other | F(28,257)=1.290, p=0.1565 (NS), multiple $R^2$ = 0.1232, adjusted $R^2$ = 0.02771 | |
| QCHAT | F(28,257)=1.488, p=0.05927 (NS), multiple $R^2$ = 0.1395, adjusted $R^2$ = 0.0457 | |

Multiple linear regressions to predict each outcome measure, given the 11 RSNs, birth status, PMA at scan, sex, total number of FD outliers and network density in the combined cohort of term-born (n=300) and preterm-born (n=66) infants.
